# Measurement of Private Equity Ownership in Healthcare Research: Review and Opportunities

**DOI:** 10.1093/geroni/igaf122.1404

**Published:** 2025-12-31

**Authors:** Sean Huang, Jennifer Bunker, Cassandra Hua, Yashaswini Singh, Momotazur Rahman, Kali Thomas

**Affiliations:** Georgetown University, Washington, District of Columbia, United States; Johns Hopkins University, Baltimore, Maryland, United States; University of Massachusetts Lowell, Lowell, Massachusetts, United States; Brown University School of Public Health, Providence, Rhode Island, United States; Brown University, Providence, Rhode Island, United States; Johns Hopkins University, Baltimore, Maryland, United States

## Abstract

Because ownership reporting requirements vary by healthcare entity types, PE ownership may not be publicly reported and researchers must rely on varying sources to measure PE ownership. We aim to understand measurement considerations relevant for examining PE in health care, including how researchers operationalize PE ownership of healthcare organizations, and to what extent the heterogeneity of private equity transaction (e.g., by boutique vs. general PE firms, different deal, or amount of debt financing) is studied in the literature. We systematically reviewed quantitative studies focused on PE and healthcare providers in the US published in years 2008-2023. We find that proprietary commercial databases and news/web searches are the two major approaches researchers used to identify private equity transitions. About 48% of the papers rely on commercial databases, and 14% of the papers use news releases and web searches to identify PE transitions. Additional 39% use a combination of commercial databases and news searches to construct their samples. Most papers often rely on a binary indicator of PE ownership, but do not study the heterogeneity effects of PE transitions. Overall, we find researchers increasingly rely on multiple commercial datasets to identify private equity transitions. However, most of the literature doesn’t discuss the potential discrepancy of PE definitions among data vendors. A more transparent and uniform database of PE and non-PE transactions, can facilitate studies on understanding nuance and heterogeneity that can facilitate a better design and regulation of PE transitions for all sectors of healthcare, including assisted living.

